# Effects of voluntary running on the skeletal muscle of rats with pulmonary artery hypertension

**DOI:** 10.3389/fphys.2023.1206484

**Published:** 2023-07-04

**Authors:** Filipe Rios Drummond, Leôncio Lopes Soares, Tiago Ferreira Leal, Luciano Bernardes Leite, Leonardo Mateus Teixeira Rezende, Meilene Ribeiro Fidelis, Victor Neiva Lavorato, Denise Coutinho Miranda, Miguel Araújo Carneiro-Júnior, Mariana Machado Neves, Luciane Carla Alberici, Emily Correna Carlo Reis, Clovis Andrade Neves, Antônio José Natali

**Affiliations:** ^1^ Department of General Biology, Laboratory of Structural Biology, Federal University of Viçosa, Viçosa, Brazil; ^2^ Department of Physical Education, Laboratory of Exercise Biology Federal University of Viçosa, Viçosa, Brazil; ^3^ Department of Physical Education, Governador Ozanam Coelho University Center (UNIFAGOC), Ubá, Minas Gerais, Brazil; ^4^ Faculty of Pharmaceutical Sciences of Ribeirão Preto, University of Sao Paulo, Ribeirão Preto, Brazil; ^5^ Veterinary Department, Federal University of Viçosa, Viçosa, Brazil

**Keywords:** voluntary running, physical effort, skeletal muscle, monocrotaline, gene expression

## Abstract

The effects of voluntary running on the skeletal muscle of rats with pulmonary arterial hypertension (PAH) were tested in the present study. PAH was induced in rats by a single injection of monocrotaline (MCT, 60 mg/kg). Rats in the sedentary hypertension (HS) group had their tolerance to physical exertion reduced throughout the experiment, while those in the sedentary control (SC), exercise control (EC), exercise hypertension (EH) and median exercise (EM) groups maintained or increased. Despite that, the muscular citrate synthase activity was not different between groups. The survival time was higher in the EH (32 days) than in the SH (28 days) (*p* = 0.0032). SH and EH groups showed a lower percentage of muscle fiber and a higher percentage of extracellular matrix compared to control groups (*p* < 0.0001). However, the EM and EH groups presented higher percentage of muscle fiber and lower percentage of extracellular matrix than SH group (*p* < 0.0001). Regarding muscular gene expression, the SH and EM groups showed a lower expression of PGC1-α (*p* = 0.0024) and a higher expression of VEGF (*p* = 0.0033) compared to SC, while PGC1-α was elevated in the EH. No difference between groups was found for the carbonylated protein levels (*p* > 0.05), while the TNF-α/IL-10 ratio was augmented in the EH (*p* = 0.0277). In conclusion, voluntary running augments the proportion of fiber and affects the gene expression of inflammatory and mitochondrial biogenesis’ markers in the skeletal muscle of rats with MCT-induced PAH, which benefits their survival and tolerance to physical effort.

## 1 Introduction

Pulmonary artery hypertension (PAH) is caused by a progressive increase in pulmonary vascular resistance that results in adverse right ventricular remodeling, dysfunction, and heart failure (Bernardo et al., 2020; Zolty, 2020; Humbert et al., 2022). As the disease progresses, the stability of cardiac pump and right ventricular function is impaired (Benoist et al., 2012; Fowler et al., 2015), making it difficult to manage the increased contractile demands.

In PAH, the reduction in cardiac output and consequent decrease in the availability of oxygen and nutrients in the peripheral systems, causes loss of muscle mass and leads to intolerance to physical effort (Taylor et al., 2020). Therefore, it is well established that PAH induces skeletal muscle atrophy (Batt et al., 2014; Moreira-Gonçalves et al., 2015) and dysfunctions (Riou et al., 2020). It is noteworthy that muscle waste in PAH is related to high levels of pro-inflammatory cytokines, compromised activation of pathways of protein synthesis and accumulation of dysfunctional mitochondria (Plant et al., 2010; Toth et al., 2011; Romanello & Sandri, 2013). Altogether, these changes result in diminished tolerance to physical exercise and reduced functional capacity in PAH patients, negatively affecting their quality of life and survival prognosis (McGoon et al., 2013; Zafrir, 2013; Sahni et al., 2015).

Regular aerobic exercise has been proven to be a good non-pharmacological therapy enhancing functional capacity, physical effort tolerance, ventilatory efficiency and overall cardiac function in PAH patients (Buys et al., 2015; Babu et al., 2016), which results in life quality improvement. The underlying mechanisms of such exercise benefits, however, are not well determined, especially concerning skeletal muscles, since few studies on experimental models of PAH have evaluated the effect of exercise regimens (e.g., Moreira-Gonçalves et al., 2015; McCullough et al., 2020; Vieira et al., 2020). Despite the lack of studies, in a recent review (Drummond et al., 2023), our group showed that aerobic exercise training can provide protection and reduce muscle atrophy, increase muscle capillarization and improve mitochondrial function. Nevertheless, we pointed out that studies on skeletal muscle’s structural, cellular and molecular adaptations to exercise are crucial for the development of effective preventive and/or therapeutic approaches.

Therefore, in the present study we sought to test the effects of voluntary running (i.e., intermittent-exercise regime) on the skeletal muscle of rats with pulmonary artery hypertension (PAH). We chose spontaneous wheel running since it can delay the onset of heart failure, has minimal intervention by the investigator, and is a less stressful exercise mode for the animal (Seo et al., 2014; Natali et al., 2015).

## 2 Methods

### 2.1 Animals

Forty male Wistar rats (226.7 ± 24.98) were given free access to a running wheel individually (nominated as exercised animals, E) or had no access to the wheel (nominated as sedentary animals, S). Animals were introduced to the running wheels 2 days before the injection of monocrotaline (MCT) or saline (See Soares et al., 2019). In addition, the animals received water and food *ad libitum* and were admitted to a light/dark cycle of 12/12 h.

**FIGURE 1:**
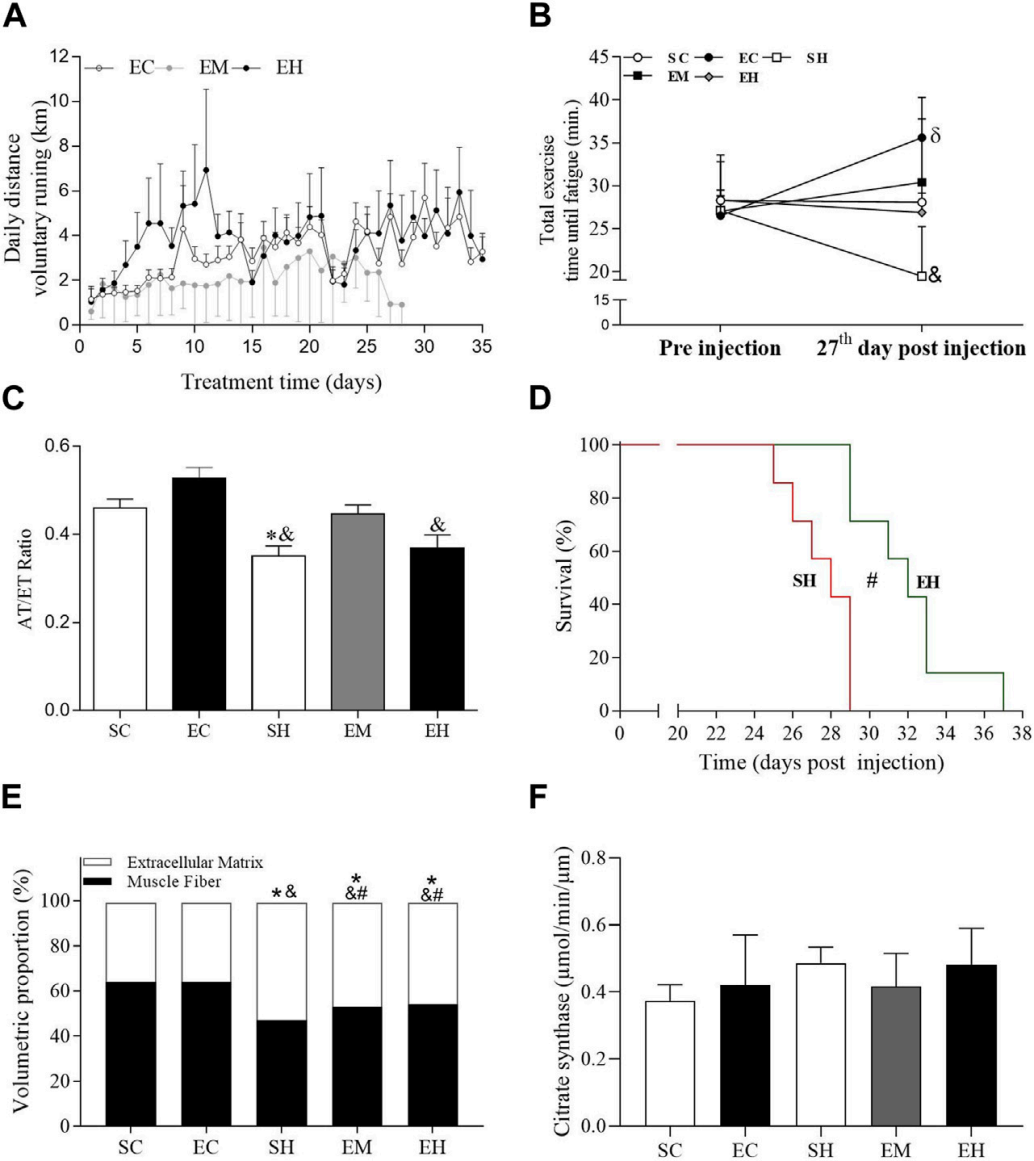
General data. **(A)** Traveled distance of voluntary running. **(B)** Total exercise time until fatigue. **(C)** Ratio of flow acceleration time to ejection time (AT/ET) in the pulmonary artery measured 25 days after injection of monocrotaline. **(D)** Survival, measured in days of onset of heart failure signs. **(E)** Proportions of myocytes and extracellular matrix in the gastrocnemius. **(F)** Citrate synthase activity in the soleus. Data are mean ± SD of 6 rats in each group for panels A, B, C and F; 8 rats in each group for panel D; and 8 images per animal in each group of 6 rats for panel **(E)**. SC, sedentary control; EC, exercised control; SH, sedentary hypertension; EH, exercised hypertension; EM, exercised median. ^*^ *P* ˂ 0.05 vs. SC; ^#^ *P* ˂ 0.05 vs. SH; ^&^ *P* ˂ 0.05 vs. EC. One-way repeated measures ANOVA followed by the Tukey *post hoc* test to compare representative days 1, 8, 15, and 22 (Panels A); One-way ANOVA followed by the Tukey *post hoc* test for between group comparisons (Panels B, C, E and F). Kaplan-Meier Curve, followed by the Log-rank *post hoc* test (Panel D).

Animals received either a single intraperitoneal injection of 60 mg/kg MCT to induce PAH (H) or an equivalent volume of saline as control (C), as previously described (Soares et al., 2019). Rats were euthanized upon showing signs of heart failure (e.g., ≥10 g weight loss overnight and/or dyspnea, cyanosis, piloerection, and lethargy) or on equivalent days for control animals. Thus, four experimental groups were configured (SC, EC, SH, EH—n = 8 for each one). In addition to these four groups, a fifth group of rats was given free access to running wheels and the same dosage of MCT, though euthanized at the median survival time day (±1 day) of SH animals for temporal comparison. This group was nominated as exercised median (EM–n = 8). The median survival time for SH and EH groups represented the time after MCT injection when more than 50% of the group showed signs of heart failure (see Figure 1D).

Regarding the exercise, the number of spins was recorded using digital magnetic counter (Cycle Computer-AS820—Assize (Machine Motors LTD; Camaquã–RS, Brazil), which allowed the calculation of the daily distance traveled and maximum running speed.

### 2.2 Whole animal evaluations

The echocardiographic evaluations were performed, as previously described (Sahn et al., 1978; Soares et al., 2019), on the 25th day after MCT application to confirm the MCT effect on the pulmonary artery.

Briefly, the images were obtained while the animals remained in the lateral decubitus position. Echocardiographic examinations were performed using the MyLabTM30 ultrasound system (Esaote, Genoa, Italia) and 11 MHz nominal frequency transducers. Pulmonary artery flow was obtained by pulsatile Doppler. The acceleration (AT) and ejection (ET) times in the pulmonary artery were evaluated and its ratio (AT/ET) was calculated. The images were collected according to the recommendations of the American Society of Echocardiography and stored for further analysis (Sahn et al., 1978).

The total exercise time until fatigue (TTF) was assessed as described earlier (Soares et al., 2019) and used as an index of physical effort tolerance. The TTF was performed before and on the 27th day after the application of MCT in all groups. Briefly, the animals started the exercise at 5 m/min, 0° inclination. Increases of 3 m/min were made every 3 min until fatigue. Fatigue was determined and the test was interrupted when the animal could no longer keep pace with the speed of the treadmill and remained for 10 s on the grid of the treadmill rather than run.

### 2.3 Histological and molecular evaluations

Following euthanasia by decapitation, the gastrocnemius and soleus muscles were excised, blotted dry, and weighed. Experiments were carried out in accordance with the Guide for the Care and Use of Laboratory Animals, and local ethical approval was obtained (Protocol number 14/2017).

Fragments of gastrocnemius were fixed in 10% formaldehyde for 48 h. Subsequently, the fragments were dehydrated in ethyl alcohol 80%, 90%, 95% and absolute for 30 min each, and kept in paraffin for 24 h. After that, the samples were included in paraffin with a hardener and stored in the oven at 60°C, for 48 h. The histological analyses of the gastrocnemius were carried out in sections of 5 μm thickness stained with hematoxylin and eosin, as described by others (Wang et al., 2018). The proportion of muscle fiber and extracellular matrix in the gastrocnemius was assessed using a grid with 130 intersections in Image-Pro Plus 4.5 software (Media Cybernetics, Silver Spring, MD, United States) projected onto eight random images per animal (total of 1,040 points per animal). Coincident points were registered in the fibers and extracellular matrix, then the percentage of points in each component was calculated.

Biomarkers of oxidative stress and metabolism were evaluated in the soleus. The concentration of carbonyl protein was detected spectrophotometrically by derivatizing the carbonyl group with 2, 4-dinitrophenylhydrazine (DNPH); and the absorbance was normalized by the quantification of the protein using the Bradford ^®^ reagent, as described earlier (Reznick and Packer, 1994). The citrate synthase activity was determined by the quantification of the complex formed between the released CoA and the 5, 5′-Dithiobis-(2-nitrobenzoic acid) (DTNB) in the medium, following protocol adapted by Alp et al. (1976).

The gene expression of biomarkers for mitochondrial biogenesis (peroxisome proliferator activated receptor co-activator-1α [PGC-1α]), angiogenesis (vascular endothelial growth factor [VEGF]) and inflammation (tumor necrosis factor α [TNF-α] and interleukin 10 [IL-10]) were quantified in the gastrocnemius by using the real-time polymerase chain reaction (qRT-PCR) technique, as described by Livak and Schmittgen (2001). The following specific primers (Thermo Fisher Scientific, EUA) were used: β-actin (forward AGCCATGTACGTAGCCAT and reverse CTC​TCA​GCT​GTG​GTG​GTG​AA), PGC-1α (forward ACC​CAC​AGG​ATC​AGA​ACA​AAC​C and reverse GAC​AAA​TGC​TCT​TTG​CTT​TAT​TGC), VEGF (forward GTA​CCT​CCA​CCA​TGC​CAA​GT and reverse GCA​TTA​GGG​GCA​CAC​AGG​AC), TNF-α (forward TGG​GCT​ACG​GGC​TTG​TCA​CTC and reverse GGG​GGC​CAC​CAC​GCT​CTT​C) and IL-10 (forward GAA​GGA​CCA​GCT​GGA​CAA​CAT and reverse CCT​GGG​GCA​TCA​CTT​CTA​CC).

### 2.4 Statistics

Survival was tested using the Kaplan-Meier curve followed by the *post hoc* Log-rank test. Initially, a Shapiro-Wilk normality test was performed. Subsequently, running distances (on representative days 1, 8, 15, and 22) were compared by two-way repeated measures analysis of variance (ANOVA) and cellular, biochemical, molecular, body weight and muscle weight parameters were compared by one-way ANOVA or Kruskal–Wallis. ANOVAs were followed by Tukey’s *post hoc* test and Kruskal–Wallis by Dunn’s *post hoc* test for multiple comparisons. *P* ˂ 0.05 was considered statistically different. The number of rats used in each experiment is shown in the legends of the tables and figures. All analyzes were performed using the statistical program GraphPad Prism 8.0^®^.

## 3 Results

Rats injected with MCT or saline solution were able to run voluntarily throughout the experimental period (Figure 1A). There was no significant difference between groups on the average daily running distance on representative days (days 1, 7, 14, 21, and 28). Regarding physical effort tolerance, the mean TTF was not different between groups before MCT injection (Figure 1B). However, on the 27th day after MCT injection, rats from the SH group reduced the TTF, which was significantly lower compared to those rats in the EC animals (*p* < 0.0001). Furthermore, the EC group increased the TTF on day 27 post injection compared to pre injection (*p* = 0.0055). The rats from the other groups maintained the tolerance to physical effort. MCT significantly reduced the AT/ET ratio in the SH group compared to control rats in the SC and EC groups (*p* < 0.05), while those in the EM group showed an intermediate value, because it does not differ from the control and SH groups (Figure 1C). The AT/ET ratio of rats in the EH group was lower than that in those rats from the EC group. Although the rats in the SH and EH groups displayed signs of heart failure, the median survival time in EH rats (32 days) was significantly longer than that in SH animals (28 days) (Figure 1D), indicating a benefit of exercise. Rats in the SH, EH, and EM groups had a lower percentage of muscle fiber and a higher percentage of extracellular matrix in the gastrocnemius, compared to those in the SC and EC groups (Figure 1E). However, rats from the EM and EH groups had a higher percentage of muscle fiber and a lower percentage of extracellular matrix compared to those from SH group (*p* = 0.0255 and *p* = 0.0196, respectively). Regarding the soleus muscle aerobic metabolism, no significant difference was observed between the groups in the activity of citrate synthase (Figure 1F).

**FIGURE 2:**
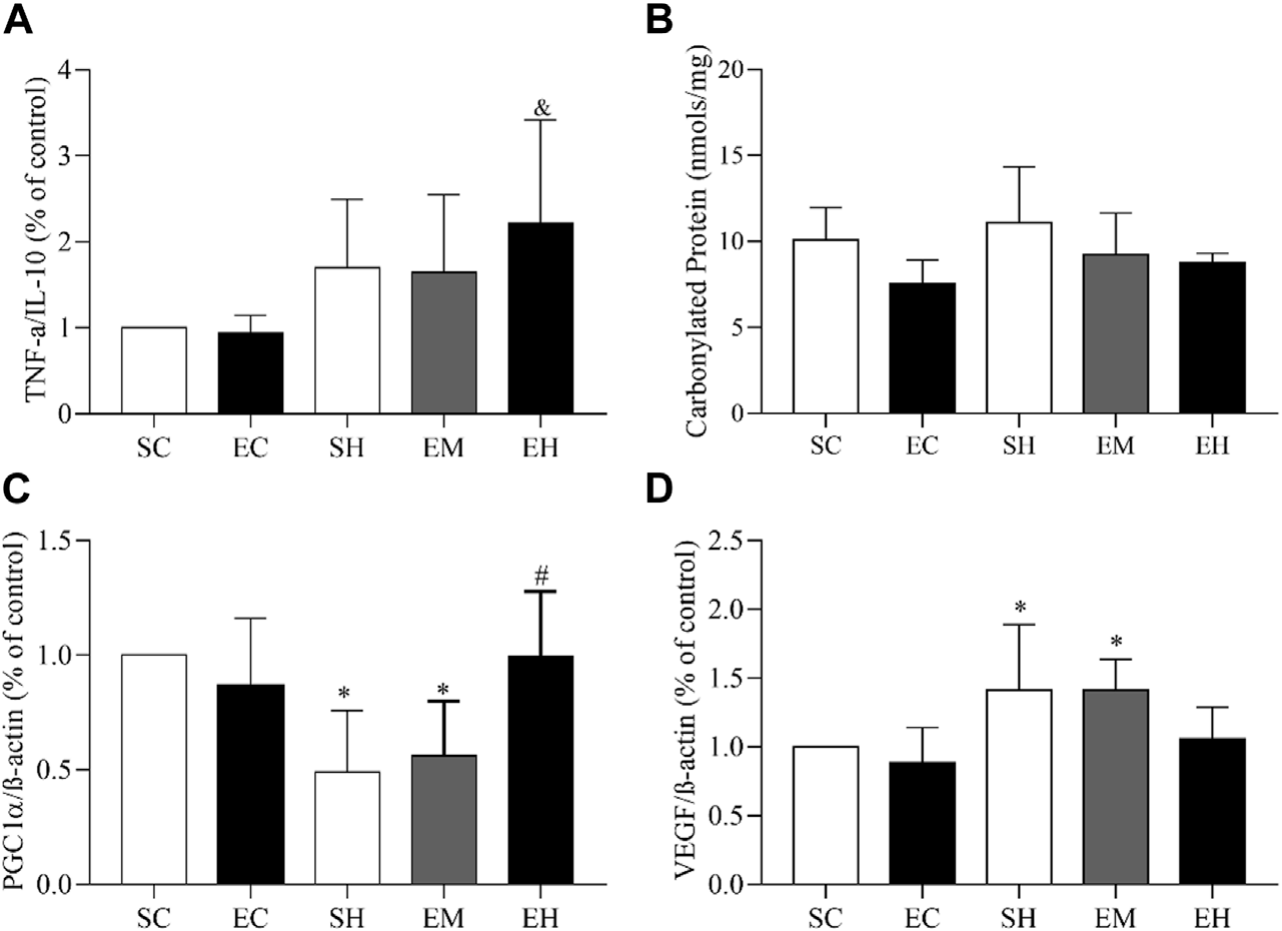
Gene expression and oxidative stress in skeletal muscles. **(A)** TNF-α/IL-10 ratio in the gastrocnemius. **(B)** Carbonyl protein concentration in the soleus. **(C)** PGC1-α/β actin ratio in the gastrocnemius. **(D)** VEGF/β actin ratio in the gastrocnemius. Data are mean ± SD of 6 rats in each group. SC, sedentary control; EC, exercised control; SH, sedentary hypertension; EH, exercised hypertension; EM, exercised median. ^*^ *P* ˂ 0.05 vs. SC; ^#^ *P* ˂ 0.05 vs. SH; ^&^ *P* ˂ 0.05 vs. EC. One-way ANOVA followed by the Tukey *post hoc* test for between group comparisons.

Regarding the gene expression of muscle inflammatory markers, an increase in the TNFα/IL-10 ratio was observed in the gastrocnemius of the EH group, compared to the EC group (*p* = 0.0429) (Figure 2A). As for the muscular oxidative stress, the concentrations of carbonylated protein in the soleus were not statistically different between groups (Figure 2B). Regarding muscular mitochondrial function and angiogenesis, rats from the SH and EM groups showed lower gene expression of PGC1α (*p* = 0.0024) (PGC1α/β-actin; Figure 2C) and higher gene expression of VEGF/β-actin (VEGF/β-actin; Figure 2D) in the gastrocnemius (*p* = 0.0033), compared to those in the SC group.

## 4 Discussion

In the present study, we used a model of MCT-induced PAH to examine the effects of voluntary running on the skeletal muscle. MCT was found to increase the pulmonary artery resistance (i.e., reduced AT/ET). In addition, at the skeletal muscle morphometric level, MCT increased the proportion of extra cellular matrix and reduced the amount of muscular fiber. Despite these changes, the muscular citrate synthase activity, TNF-α/IL-10 ratio, and the carbonylated protein levels were not different between groups. Furthermore, MCT reduced the muscular gene expression of PGC1-α and increased the gene expression of VEGF. No difference between groups was found for the TNF-α/IL-10 ratio and the carbonylated protein levels. Such MCT effects negatively impacted the survival and physical effort tolerance (i.e., reduced TTF) of the animals.

More importantly, we demonstrated that voluntary running performed during the development of PAH prevented the increase in pulmonary artery resistance and the proportion of extracellular matrix, which resulted in increased physical effort tolerance and survival.

Regarding tolerance to physical exertion, the EM and EH groups maintained similar performance to the SC group at the end of the intervention period. Therefore, physical exercise contributed to attenuate the reduction in the percentage of muscle fibers, which may help maintain the structure and function of the skeletal muscle. It is believed that the antifibrotic effect of physical exercise is related to its anti-inflammatory properties, since exercise reduces the amount of pro-inflammatory cytokines that induce skeletal muscle proteolysis (Agarwal et al., 2009). The increase in muscle fiber percentage observed in exercised hypertensive rats (EH and EM) reinforces the results reported by others (Moreira-Gonçalves et al., 2015) in rats submitted to continuous running on a treadmill performed both before and after MCT injection. Moreover, there are evidence that aerobic exercise training either continuous (Moreira-Gonçalves et al., 2015; Silva et al., 2021) or intermittent (Natali et al., 2015; Soares et al., 2019) can improve cardiac structure and function; and that pulmonary vascular reactivity (Kashimura et al., 1995) and efficiency in pulmonary gas exchange (Favret et al., 2006) are benefited by aerobic exercise. These factors may also have contributed to the increased tolerance to physical exertion observed in our study.

In the present study, the inflammatory state of skeletal muscle was evaluated through the TNF-α/IL-10 ratio. TNF-α is a cytokine, produced mainly by macrophages, but it is also produced by other muscle cells inducing various cellular responses, such as apoptosis, proliferation and production of inflammatory molecules (Wei et al., 2008). IL-10, on the other hand, is known for its anti-inflammatory effect, protecting skeletal muscle fibers from the actions of TNF-α and IL-6 in insulin metabolism. The results of the present study show that there was only an increase in the inflammatory state in the EH group, which is an unexpected result. Benatti and Pedersen (2015) showed that myokines can induce an acute anti-inflammatory response after each exercise session. According to Steensberg et al. (2003), when myokines such as IL-6 are released into the bloodstream, there is the induction of a subsequent increase in the production of IL-1 agonist receptor (IL-1ra) and IL-10 by mononuclear leukocytes in the blood, thus generating an indirect anti-inflammatory. Therefore, the increase in the inflammatory state may be an attempt to generate an indirect anti-inflammatory response.

Regarding the gene expression of PGC1-α, it was decreased in the SH and EM groups. PGC1-α is a co-activator protein that binds to several transcription factors, leading to increased expression of genes involved in mitochondrial biogenesis and increased respiratory function (Finck and Kelly, 2006). Our voluntary running regime increased the gene expression of PGC1-α (EH group), however, in animals from the EM group such effect was not present. The arrival of oxygen in the active muscles can be impaired in animals with PAH (Marra et al., 2015), and this fact may explain the increased expression of PGC1-α in the animals from the EH group and not in those from the EM group, since the animals in the EH group were in a more critical stage of the disease.

No differences between groups were observed in the muscular oxidative metabolism (e.g., citrate synthase activity in the soleus). A possible explanation for the absence of change of citrate synthase activity is the large number of red fibers (approximately 95%) in the soleus (Medeiros et al., 2000), since glycolytic muscle fibers suffer more deleterious effects of PAH (Wüst et al., 2012). This fact can also explain the absence of carbonylated proteins between groups. Due to its characteristic, the soleus muscle is less susceptible to oxidative stress (Wüst et al., 2012). On the other hand, it is known that aerobic physical exercise increases the activity of citrate synthase in the soleus muscle (Medeiros et al., 2000), which did not occur in the present study. A possible explanation is the fact that voluntary running is an intermittent exercise of relatively high intensity, and this does not generate the expected adaptations of an continuous low-intensity aerobic exercise. Furthermore, the training time was relatively short, which may also explain the lack of increase in citrate synthase enzyme activity.

The muscular gene expression of VEGF was also augmented in the SH and EM groups. Such change seems to be a compensatory mechanism to avoid further muscular damages in response to the lack of O_2_ in the microcirculation, since the skeletal muscle in PAH patients seems to be impaired, evidenced by the low O_2_ saturation (Dimopoulos et al., 2013). Our results indicate that voluntary running reduced the VEGF gene expression by the end of the experiment in the EH group, but not in the EM group. Although it must be considered that the rats in the EH group were in a more critical stage of the disease, further studies are needed to help explain such an exercise effect, since continuous treadmill running increased VEGF gene expression in the lung of rats with MCT-induced PAH (Colombo et al., 2016).

The prolonged survival displayed by the EH group reinforces the results of previous studies reported by our group (Natali et al., 2015; Soares et al., 2019). Apart from the skeletal muscle adaptations, other factors, such as improvements in the structure (Moreira-Gonçalves et al., 2015; Soares et al., 2018) and function of the myocardium (Natali et al., 2015; Soares et al., 2018), thus favoring cardiac output.

A limitation of the study was the failure to carry out analysis of the cross-sectional area, capillary density and histochemical assays in the skeletal muscle of the animals, which could more consistently support the findings on morphometric changes and add important information about the effects of PAH on muscle structure and the possible protective effects of exercise.

In conclusion, voluntary running increases the proportion of fiber and affects the gene expression of inflammatory and markers of mitochondrial biogenesis in the skeletal muscle of rats with MCT-induced PAH, benefiting their survival and tolerance to physical effort.

## Data Availability

The original contributions presented in the study are included in the article/Supplementary Materials, further inquiries can be directed to the corresponding author.
